# Medical Practitioners and Dentists

**Published:** 1887-09

**Authors:** 


### Medical Practitioners and Dentists.

A question is raised by a correspondent of the *Journal of the British Dental Association* as to the propriety or taste of medical practitioners charging full fees where they are asked to attend dentists. Our opinion is strong that medical men, who owe so much to dentists, will find it highly agreeable to be of any service in return. Who that has had a tooth saved, or even a tooth skilfully extracted, and who knows the extent to which dentists are consulted by medical men and their families, and the unfailing kindness which is experienced in this way at their hands, will have a doubt that in treating dentists as we should treat medical men we are acting on the best instincts of human nature, as well as on the best traditions of the profession? We have, however, always maintained that dentists should hold a surgical as well as a dental qualification; in which case the question need not have been raised.—*The Lancet.*

